# Investigating the role of circulating tumor cells in gastric cancer: a comprehensive systematic review and meta-analysis

**DOI:** 10.1007/s10238-024-01310-6

**Published:** 2024-03-30

**Authors:** Mohammad Reza Eskandarion, Sharareh Eskandarieh, Sara Tutunchi, Abbas Shakoori Farahani, Reza Shirkoohi

**Affiliations:** 1 Cancer Research Center, Cancer Institute, IKHC, Tehran University of Medical Sciences, Tehran, Iran; 2 Multiple Sclerosis Research Center, Neuroscience Institute, Tehran University of Medical Sciences, Tehran, Iran; 3 Department of Medical Genetics, School of Medicine, Shahid Beheshti University of Medical Sciences, Tehran, Iran; 4 Medical Genetics Ward, IKHC Hospital Complex, Tehran University of Medical Sciences, Tehran, Iran

**Keywords:** Circulating tumor cells, Gastric cancer, Prognosis, Diagnostic power, Meta-analysis

## Abstract

Investigating the role of circulating tumor cells (CTCs) and their characteristics is still controversial in patients with gastric cancer (GC). Therefore, in this study, to provide a comprehensive review and meta-analyses of the literature on association of CTCs with gastric cancer, Scopus, Web of Science, Embase, and Medline were searched for systematic reviews and meta-analyses conducted during February 2022 using the keywords. Risk of bias, hazard ratios (HRs), and risk differences (RD) were assessed. Forty-five studies containing 3,342 GC patients from nine countries were assessed. The overall prevalence of CTC in GC was 69.37% (60.27, 77.78). The pooled result showed that increased mortality in GC patients was significantly associated with positive CTCs, poor overall survival (HR = 2.73, 95%CI 2.34–3.24, *p* < 0.001), and progression-free survival rate (HR = 2.78, 95%CI 2.01–3.85, *p* < 0.001). Subgroup analyses regarding markers, detection methods, treatment type, presence of distance metastasis, presence of lymph node metastasis, and overall risk of bias showed significant associations between the groups in terms of the incidence rates of CTCs, OS, and PFS. In addition, the results of risk differences based on sampling time showed that the use of the cell search method (RD: − 0.19, 95%CI (− 0.28, − 0.10), *p* < 0.001), epithelial marker (RD: − 0.12, 95%CI (− 0.25, 0.00), *p* 0.05) and mesenchymal markers (RD: − 0.35, 95%CI (− 0.57, − 0.13), *p* 0.002) before the treatment might have a higher diagnostic power to identify CTCs and also chemotherapy treatment (RD: − 0.17, 95%CI (− 0.31, − 0.03), *p* 0.016) could significantly reduce the number of CTCs after the treatment. We also found that the risk differences between the clinical early and advanced stages were not statistically significant (RD: − 0.10, 95%CI (− 0.23, 0.02), *P* 0.105). Also, in the Lauren classification, the incidence of CTC in the diffuse type (RD: − 0.19, 95%CI (− 0.37, − 0.01), P0.045) was higher than that in the intestinal type. Meta-regression analysis showed that baseline characteristics were not associated with the detection of CTCs in GC patients. According to our systematic review and meta-analysis, CTCs identification may be suggested as a diagnostic technique for gastric cancer screening, and the outcomes of CTC detection may also be utilized in the future to create personalized medicine programs.

**Supplementary Information:**

The online version contains supplementary material available at 10.1007/s10238-024-01310-6.

## Introduction

Gastric cancer (GC) can be considered as the sixth most frequently diagnosed cancer (5.7%) and the third leading cause of cancer deaths (8.2%) globally [1]. Since GC is frequently asymptomatic or may associate with mild non-specific gastrointestinal symptoms in its early stages, it is often diagnosed in the advanced stages, and consequently, no therapeutic or survival benefit is obtained from conventional surgery or chemo/radiotherapy [2]. More than 40% of GC patients do not show any response to chemotherapy and the rest resist chemotherapy, resulting in a low survival rate and the limitation of treatment options [3]. Therefore, patients with primary GS undergo endoscopy, removal or gastrectomy with lymphadenectomy, and also systemic chemotherapy is prioritized as a standard treatment in patients with non-removable or recurrent gastric cancer [4]. Moreover, the advanced endoscopic methods, including chromoendoscopy [5] and narrow-band imaging (NBI) [6], are considered as more reliable tools for the detection of GC as compared with conventional diagnostic tools. However, their use is limited due to invasiveness and concerns on their cost [6]. Serum tumor markers, including carcinoembryonic antigen (CEA) and CA-125 and CA-19–9 carcinoma antigen, are also commonly used for management of the GC patient. However, they are not sufficient to detect the disease and determine the prognosis for patients with GC [7]. Some studies have also demonstrated that clinical manifestations, radiological evaluations, and serum tumor markers may not able to provide sufficient information on the onset of metastasis or predict the clinical outcomes from GC using high-reproducibility and high-accuracy method. To improve cancer survival rates, it is therefore necessary to develop the novel diagnostic techniques, which not only can provide accurate predictors of the time of possible metastasis formation, but also monitor the disease and evaluate the efficacy of anti-tumor therapies.

Recently, new methods with various features, including non-invasive and fast, high accuracy and sensitivity, have been developed. Circulating tumor cells (CTCs) was first introduced in 1889, which are the primary tumor cells successfully passing metastasis process and can migrate throughout the body with entering the bloodstream [8]. There are also several studies on the use of CTCs in different cancers (such as colon cancer, breast cancer, non-small-cell lung cancer, head and neck squamous cell) [9–14], and also their role has been proven. However, studies conducted on GC show that there is still controversy about the identification of CTCs in GC considering the availability of different techniques for the introduction of appropriate diagnostic methods and markers [15]. Also, some studies have pointed out the presence of CTCs in malignant conditions of GC and reported that their investigation is helpful in different conditions of disease, while some have concluded that due to the sensitivity of diagnostic tests for CTCs and their small number in the metastatic stage, they cannot be used for screening in GC [16]. Another important feature of CTCs is their application in treatment, one of the most important of which is the counting of CTCs so that by counting their exact number we can diagnose both the stage of the disease and the survival time of the patient and also monitor the treatment. Thus, the incidence of CTCs in different phases of treatment and the effects of drugs on the patient's treatment can be evaluated [17, 18]. However, there is controversy about studies conducted on the most suitable stage for evaluating the role of CTCs in GC. Also, in the discussion of target therapy, in which by identifying a biomarker and tracing it in CTCs a specific drug can be obtained for the disease, as a clear manifestation of personalized medicine, there are still different results [19].

Therefore, the role of CTCs in the detection of GC patients has been extensively studied and debated. It is evident that conducting a systematic review and meta-analysis on the role of CTC in the early diagnosis of gastric cancer and a review of information on the stages of this disease and introduction of treatment targets by identifying markers and investigating appropriate diagnostic methods can be useful to identify both CTCs in the detection of GC and treatment strategies. Consequently, the quality of life of these patients is promoted and also it would be effective for resource allocation by health authorities. There are few systematic reviews and meta-analyses performed on the role of (CTCs) in the diagnosis of patients with GC [20–26], but all of them specifically point out only one of the related topics discussed above and also there is no comprehensive meta-analysis study in this regard. Also, the latest meta-analysis published in this regard is related to Yunhe Gao's 2019 [24]. Therefore, the aim of this study was to perform a systematic review and meta-analysis of the benefits and challenges of diagnosing (CTCs) and their characteristics in GC patients.

## Materials and methods

### Search strategy

In this study, PRISMA (Preferred Reporting Items for Systematic Reviews and Meta-Analyses) guideline was employed to design a systematic review [27]. The keywords, including “circulating tumor cells”, “CTCs” and “gastric cancer”, were variably combined. Also, databases, including Scopus, Web of Science, Embase, and Medline were searched for systematic reviews and meta-analyses published until February 22, 2022.

### Screening and eligibility criteria

We applied databases to identify studies conducted on CTCs and GC with limiting a review of studies in English language. The titles and abstracts of all full texts identified by the databases were assessed. The studies that met the following criteria were included in the study, and also the full texts were read to confirm that they met the following criteria.

1. Studying as case controls and causes of GC.
2. Diagnosing GC in patients by a pathologist.
3. The use of CTC markers in diagnosing the disease.
4. Survival data for patients should be known.
5. In studies, statistical calculations should be presented in their research method.
6. Available in full-text form.

Studies were excluded if they met the following exclusion criteria:

1. The sample size was less than 20.
2. Samples were not taken from peripheral blood, e.g., urine or bone marrow sample
3. CTC separation methods were not mentioned in the study.
4. Patients who were eligible in other studies should not overlap.
5. Some studies did not employ any CTC separation methods and they only used CTC detection methods; therefore, all of them were excluded from this study.

Moreover, we performed a manual hand search of reference lists of main identified studies and relevant reviews to identify additional studies that could have been missed

### Extraction of the data and quality evaluation

Two expert reviewers independently investigated the summary of papers in terms of relevance with the subject, goals, and inclusion /exclusion criteria and provided the extracted data, such as first author name, year, country, the type of the marker used, year of publication, study region, number of people studied, age, gender, tumor stage, TNM stage, maker studied, methods detection, CTC incidence, follow-up duration (months), clinical therapy, blood sample volume, timing of blood sample collection, distance and lymph node metastasis, cutoff value and hazard ratios (HRs) for overall survival (OS) and progression-free survival (PFS). Forasmuch as more than one marker was detected in studies, we had divided between two groups (epithelial markers, mesenchymal markers). If an abstract did not clearly meet the eligibility criteria, the whole full-text review was performed to evaluate whether studies completely met the exclusion criteria. Studies that met the eligibility criteria were included in the study. In case of different ideas on an article and disagreement between two individuals in its inclusion, a senior reviewed the article and the idea of the third person was determinant and also duplicates on the same data were removed.

### Assessing of risk of bias in included studies

A modified Cochrane risk of bias instrument, which was scored as Yes, no, and Unknown, was employed to assess risk of bias in the included studied. That is, if all 6 questions were answered Yes, they were considered as low risk, if even one question was answered No, it was considered as high risk, and if one question was Unknown or Unclear, its bias was considered as unknown [28]. The risk of bias calculation in Cochrane consists of several parts, including “adequate qualifications,” “the measurement of equality,” “controlled confounding,” “adequate follow-up,” “free of selective outcomes,” and “other factors” [29] (Supplementary Table 1).

### Statistical analysis

All statistical analysis for the data in this meta-analysis was conducted by using the STATA version 17.0 (Stata Corp, College Station, TX, USA). The HR values with 95% confidence intervals (CIs) for OS and PFS were recorded. Also, when HR values were not given, we estimated HRs from the Kaplan–Meier curves using an HR calculation Excel spread sheet presented by Tierney et al. [30–32]. We employed Forrest plots to demonstrate the pooled HR, and also HR > 1 had the worse survival outcome. Besides, the estimated risk differences (RD) were applied to show the correlation between the presence of CTCs in sampling time and various stages. The pooled HR and RD values were combined with a 95% CI and also a *p*-value of 0.05 was considered statistically significant. Heterogeneity was evaluated by using both *I*^2^ inconsistency test and χ2-based Cochran’s *Q* statistic test [33] in which *I*^2^ > 50% or *P* < 0.1 showed a substantial heterogeneity. When the *I*^2^ value of < 50% and the *P* value of > 0.1 were observed in all the analysis, the fixed-effect model was applied, or the random effects model was conversely employed [34].

Galbraith plot was employed to assess the extent of heterogeneity, and also our meta-analysis showed substantial heterogeneity. Moreover, Begg's test and Egger's test were applied to detect the potential publication bias [35] and also the *p*-value of 0.05 was considered to be significant publication bias. Furthermore, subgroup analyses were made based on the differences of the obtained data such as the type of the marker used, clinical stage, TNM stage, maker studied, methods detection, type of treatment, time taken to collect the baseline or postoperative data, distance and lymph node metastasis, and quality of the studies. Subgroup analyses were carried out only when two or more studies were included in the subgroups and therefore were excluded any subgroup containing fewer than two studies.

Also, when the results of funnel plot and Egger’s and Begg’s tests showed a significant publication bias, we employed the nonparametric Trim-and-Fill test that HR was changed following the imputation of some studies [36]. We also performed univariate meta-regression analyses (random effects) on the same factors so as to assess the potential sources of heterogeneity.

## Results

### Baseline characteristics of included studies

**Fig. 1:**
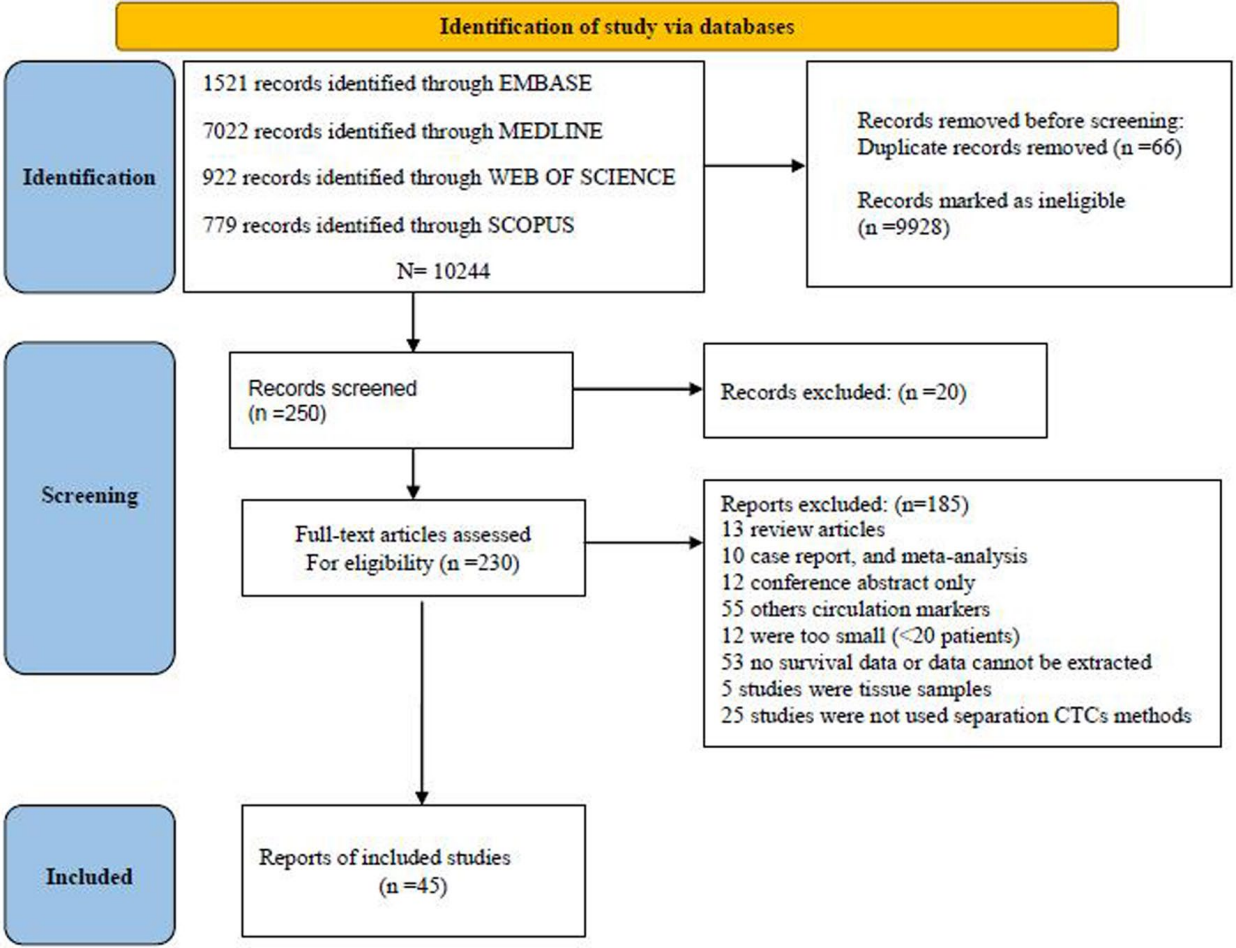
Flow chart of study selection

The systematic literature searches (Fig. 1) identified a total of 45 studies that met inclusion criteria. Table 1 shows the characteristics of 45 studies included in this study. 3,342 patients had GC cancer with a sample size range of 20 to 228 patients (median: 74 patients). The included studies were performed in 9 countries, such as China (22 articles), Japan (12 articles), Korea (3 articles), the USA (3 articles), Brazil, Poland, France, the Czech Republic, and Australia, each of which has one article published between 2009 and 2022. The median age of the patients was 61 years, and the male percentage was 70%. The median follow-up duration was 29 months and 6 articles were not reported. The pooled analysis of the descriptive variables in the 45 studies showed that the overall prevalence (%) of CTC was 69.37 (60.27, 77.78), and also, I-square was 96.57 (*P* < 0.001). The overall prevalence (%) of CTC in early stage for 21 studies was 44.48 (29.92, 59.48), in advanced stage (21 studies) was 56.17 (43.73, 68.25), in intestinal type (11 studies) was 37.65 (25.07, 51.07), in diffuse type (11 studies) was 56.32 (39.89, 72.14) and in mixed stage (21 studies) was 55.62 (27.06, 82.50). The median cutoff value obtained from 36 studies was 3.1 in 8.2cc blood. HRs for both OS in 23 studies and PFS in 19 articles for-free survival were recorded. Also, 17 articles reported both of them. Although some studies [37–42] did not report HRs, we approximated HRs from the Kaplan–Meier curves using the HR calculation Excel spreadsheet presented by Tierney et al. [30–32]. Risk.

**Table 1:** Reporting baseline characteristics in the included studies

Study/(reference)	Country of study	Sample size	Mean age	Male percent	Tumor stage	CTC cutoff N/V(ML)	Target	Detection method	Follow-up duration months	Treatment	Sampling time	CTC incidence before/after treatment	Outcomes measured	Quality
Pengjie Yu 2022/[43]	China	45	55	89	II-IV	1/10ML	EPCAM, CK8, CK18, and CK19, vimentin and twist	Can Patrol CTC enrichment technique	12	Chemo	Before treatment	95	NR	Low
Yang Chen 2021/[37]	China	111	60	83	TNM	3/6ML	KRAS	WGA	23	Chemo	Before treatment	92	OS, PFS	High
Yang Chen 2021/[38]	China	114	60	81	TNM	NA	HER2	SE-iFISH	23	Chemo	Before treatment	35	OS, PFS	High
Chengcheng Qian 2021/[44]	China	72	65	NA	I–IV	1/5ML	CD45, DAPI, and CEP8	Im-FISH, Cell Search	50.18	Chemo	Before treatment	72	OS	Low
Daisuke Matsushita 2021/[45]	Japan	61	68	85	NA	NA	HER2, Cell Search	Cell Search	50	Chemo	Before treatment	62	NR	High
Yui Ishiguro 2021/[46]	Japan	54	63	66	I–III	1/7.5ML	CD45/N-cadherin	MACS	25	Surgery	Before and after surgery	90/47	NR	Low
Yinxing Zhu 2021/[47]	China	116	64	77	I–IV	3/3.2ML	CD45, DAPI, and CEP8	Im-FISH	39	Chemo	Before and after treatment	43/27	OS	Low
Joon Hyung Jhi 2021/[42]	Korea	31	63	71	TNM	7.5/7.5 mL	DAPI/CD45/TWIST/EPCAM	FAST	14	Chemo	Before and after treatment	80/32	OS	Low
Dawei Ning 2020/[48]	China	59	57	83	I–IV	3/5ML	Cell Search	Cell Search, CTC-Biopsy systems	8	Chemo	Before treatment	27	OS, PFS	Low
Kenji Kuroda 2020/[49]	Japan	100	70	62	I–IV	5/10ML	PI, CD45, EPCAM, FGFR2	FISH/FACS	25	Surgery	Prior to surgery	50	NR	Low
Mengyuan Liu 2020/[50]	China	70	63	73	I–IV	1/1ML	VIM, PD-L1	MACS/ flow cytometry		Surgery	before Or after treatment	71	OS, PFS	Low
Baoguang Hu 2020/ [51]	China	41	64	73		2/3.2ML	CEP8 + /CD17 + /CD45 − /DAPI + , VIMENTIN, EPCAM, ULBP1	CYTTEL CTC, IMFISH	2	No Treatment	AT BASELINE	71	NR	Low
Emne A. Abdollah 2019/[39]	Brazil	55	57	60	TNM	2.8/1ML	Plakoglobin, HER2	ISET, ICC	13	surgery	Before treatment and after surgery	91/93	PFS	Low
Boran Cheng 2019/[52]	China	32	66	69	II-IV, TNM	NA	PD-L1, CD45, BD, EPCAM and CK8/18/ 19, Vimentin and Twist	Can Patrol CTC enrichment technique	NA	Chemo	Before treatment	> 80	NR	Low
Rong Lu 2019/[53]	China	42	59	69	III–IV	1/5	CA724, CA199, and CEA	ISET	5	Chemo	Before and after treatment	36/9	NR	High
Zhenlong Ye 2019/[54]	USA	45	NA	NA	III–IV	NA	CD45/EPCAM/CK18/ PD-L1/Vimentin	SE-iFISH	40	Surgery, chemotherapy, radiotherapy	Before and after treatment	96	NR	High
Yang Li 2018/ [55]	China	150	60	81	I–IV	2/3.2	DAPI + /CD45-/Chromosome multiploidy	Immunostaining-FISH	25	No Treatment	Before treatment	96	NR	High
Qiyue Zhang 2018/ [56]	China	93	45	73	I–III, TNM	5/7.5	Cell search	Cell search system	17	Surgery	Before and after surgery	33/33	OS, PFS	Low
Antoni Szczepanik 2018/[57]	Poland	228	63	66	I–IV	NA	CD45 − , cytokeratin (8, 18, and 19) and CD44	FACS	99	Surgery	Prior to surgery	13	OS	Low
Yilin Li 2018/[58]	China	115	60	NA	III–IV	2/6	(DAPI, HER2, CEP8, and CD45	SE-iFISH	31	Chemo	before and after treatment	91	NR	High
Yuji Mishima 2017/[59]	Japan	118	65	NA	NA	5/10	Cell search	3D–IF-FISH, Cell Search	24	No treatment	Before treatment	85	OS, PFS	High
Dongmei Diao 2017/[60]	China	41	61	83	III-IV	2/7.5	Cell search	Cell Search	48	Chemo	Before treatment	32	NR	High
Xiumei Zheng 2017/[40]	China	86	53	52	I–IV	2/5	CK + /Vimentin − /CD45 −	ISET, IF	13	Chemo	Before treatment	60	OS, PFS	Low
Simon Pernot 2017/[61]	France	132		53	TNM	2/7.5	Cell Search	Cell Search	24.9	Chemo	Before and after treatment	80/51	OS, PFS	Low
YONGPING LIU 2017/[62]	China	59	59	59	III–IV	2/5	ck	IF	23	Chemo	Before and after treatment	83/61	OS, PFS	Low
Daniel Brungs 2017/[63]	Australia	43	64	74	II-IV	17/7.5	Anti-EPCAM, anti-CD45, DAPI, CK	IsoFlux	NA	No treatment	Before treatment	95	OS	High
L. Zheng 2017/[64]	China	60	60	60	I–IV, TNM	NA	Size	ISET, Wright staining	23	Surgery	Prior to surgery	55	NR	High
Hwa Mi Kang 2017/[65]	Korea	116	60	64	NA	2/7.5	CK/ EPCAM/CD45// DAPI	FAST disk	12	Surgery	Prior to surgery	85	NR	High
Yilin Li 2016/ [66]	USA	31	60	68	TNM	2/7.5	DAPI, CD45, CK	SE-iFISH	10	Chemo	Before and after treatment	93/81	OS, PFS	Low
Hiroaki Ito 2016/[67]	Japan	65	59	70	NA	5/7.5	Telomerase specific	Fluorescence intensity	13	Surgery	Prior to surgery	100	OS, PFS	High
Dandan Yuan 2015/[68]	China	31	62	68	I–IV	NA	CD44, CD45	FACS	NA	Chemo	Before treatment	45	NR	High
TORU WATANABE 2015/[69]	Japan	25	73	65	I–IV	1/5	EpCAM, CD44	FACS	9	Surgery	Prior to surgery	92	NR	High
Katarina Kolostova 2015/[70]	Poland	22	69	NA	I–IV	4/8	Cytokeratin-18, Cytokeratin-19, Cytokeratin-20, ytokeratin-7, EPCAM, MUC1, HER2, EGFR	Meta cell	NA	Surgery	Prior to surgery	59	NR	High
Yilin Li 2015/ [71]	China	136	59	65	TNM	3.7/5	Cell search	Cell search	31.6	Chemo	before and after treatment	56/24	OS, PFS	High
H. Okabe 2015/[72]	Japan	136	67	51	I–IV, TNM	1.7/5	Cell search	Cell search	26	Chemo	Before treatment	18	OS, PFS	low
Su Jin Lee 2015/[41]	Korea	95	57	66	NA	5/7.5	Cell search	Cell search	8	Chemo	Before and after treatment	45/31	OS, PFS	High
Ting-Ting Li 2015/ [73]	China	44	56	70	I–IV	NA	Keratins 8, 18,19, epithelial cell adhesion molecule, Vimentin and Twist	CanPatrol TM system	17	Chemo	Before treatment	80	NR	Low
Kosei Toyoshima 2015/[74]	Japan	42	NA	71	I–IV	3/7.5	Cell search	Cell search	NA	Chemo	Before and after treatment	67	NR	High
Ilja Kubisch 2015/[75]	USA	62	64	63	III–IV	1/7.5	EPCAM, KRT19, MUC1, EPCAM, CEACAM5 and BIRC5	Immunomagnetic dynabeads, RT-PCR	17.2	Chemo	Before and after treatment	70/85	OS, PFS	Low
Study/(reference)	Region	Patient	Age	Male percent	Tumor Stage	CTC cutoff N/V(ML)	Target	Detection method	Follow-up duration months	Treatment	Sampling time	CTC incidence before/after treatment	Outcomes measured	Quality
Yilin Li 2014/[76]	China	20	NA	NA	IV	4/7.5	DAPI, CD45, CK	SE-iFISH, Cell Search		Chemo	Before and after treatment	90	NR	High
Man Li 2014/[77]	China	45	60	60	I–IV	1/10	CK19, CD44, DAPI	Immunofluorescent	30	Chemo	Before treatment	60	NR	High
M Iwatsuki 2013/[78]	Japan	34	57	73	NA	NA	Cell search	Cell search	48	No treatment	Before treatment	73	NR	High
Yoshikazu Uenosono 2013/[79]	Japan	148	64	NA	I–IV	1.7/5	Cell search	Cell search	31.6	surgery	Before and after surgery	60/10	OS, PFS	Low
Hiroaki Ito 2012/[80]	Japan	65	59	71	I–IV	5/7.5	Telomerase specific	Fluorescence microscope	20	Surgery	Prior to surgery	10/0	NR	High
Satoshi Matsusaka 2009/[81]	Japan	52	62	84	NA	4/7.5	Cell search	Cell search	29	Chemo	Before and after treatment	32/17	OS, PFS	High

N: Number; V: Volume; NR: not reported; HR: hazard ratio; OS: overall survival; PFS: progression-free survival; MSP: methylation-specific PCR; BGS: bisulfite genomic sequence; qMSP: quantitative methylation-specific PCR; NGS: next-generation sequencing; NA: Not Available

of bias assessments were performed to analyze all included studies, of which 21 studies were of low quality and 23 were of high quality and one was unclear risk (supplementary Fig. 1).

### The findings obtained from the meta-analysis conducted on incidence of CTCs and survival rates of OS and PFS

**Fig. 2:**
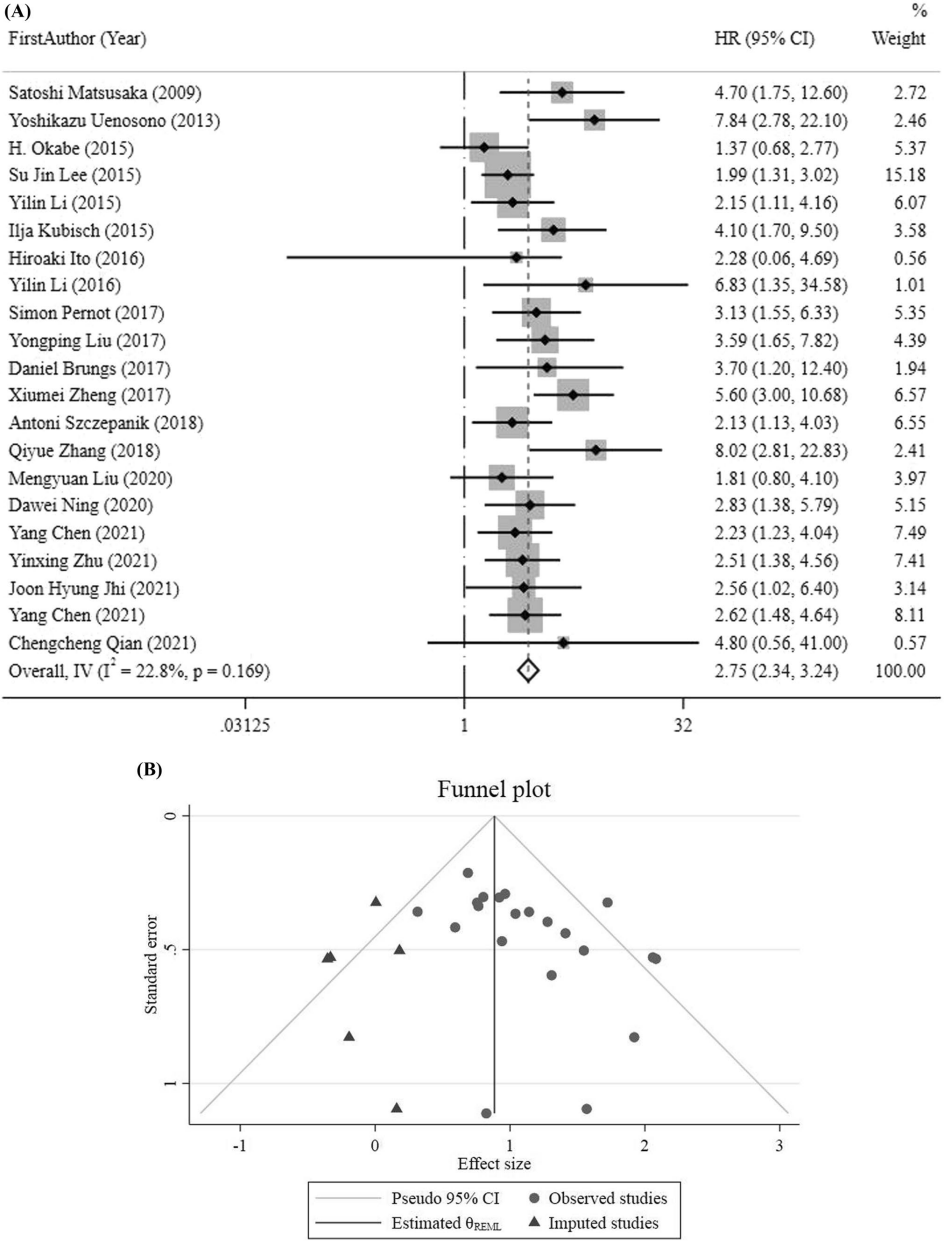

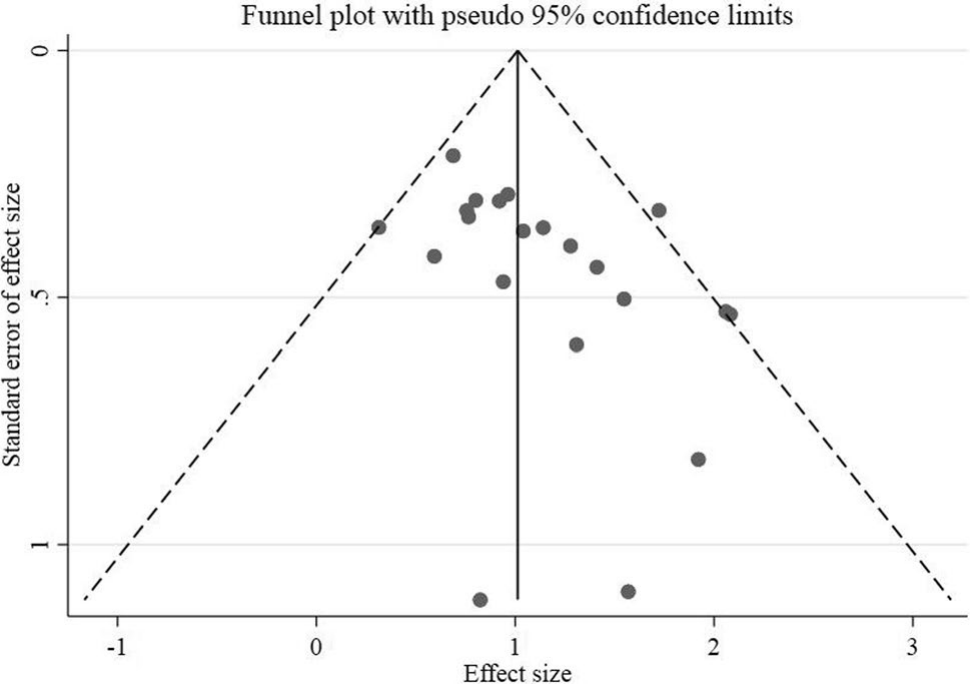
**a** HR analysis for OS. **b** OS funnel plot trim and fill. **c** Funnel plot for OS analysis

HRs for OS was obtained by meta-analysis pooling of aggregate data using the fix-effect inverse variance model used in 21 studies. Six HRs for OS were estimated from Tierney et al. [30, 37–42], and their details were provided in the Materials & Methods section. The pooled HR results demonstrated an increase in mortality rates in GC patients who had positive CTCs (HR = 2.75, 95%CI 2.34–3.24, *p* < 0.001) (Fig. 2a), indicating very low heterogeneity (*I*^2^ 22.8%, *p* = 0.169). Also, sensitivity analysis results did not show further information, but the result obtained from funnel plot and Egger’s and Begg’s tests demonstrated that there was a significant publication bias (*P*-value for Begg’s test = 0.022, *P*-value for Egger’s test = 0.025). Accordingly, the HR value (95% CI) was changed to 2.37 (2.04, 2.74) following the imputation of 6 studies in the nonparametric trim-and-fill test (Fig. 2b, c).

**Fig. 3:**
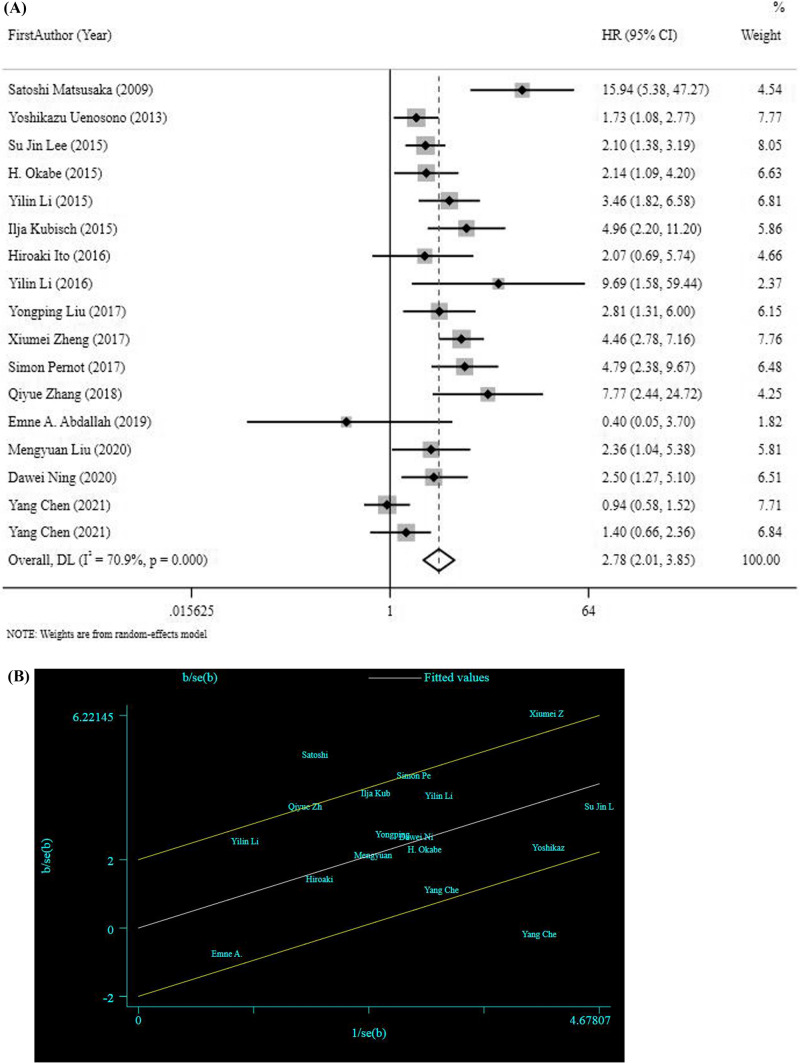
**a** HR analysis for PFS. **b** Random Gal braith plot for PFS

HRs for PFS was obtained by meta-analysis pooling of aggregate data using the random effect inverse variance model used in 17 studies. Six HRs for PFS were estimated [37–42]. The pooled HR results revealed that risk of disease progression or recurrence was significantly increased in patients with CTC positivity (HR = 2.78, 95%CI 2.01–3.85, *p* < 0.001), (Fig. 3a), indicting high heterogeneity (*I*^2^ 70.9%, *p* =  < 0.001). Moreover, the results obtained from sensitivity analysis did not show further information and also visual inspection of funnel plot and the Begg’s test (*P* = 0.187) and Egger’s test (*P* = 0.174) showed no publication bias for PFS. To identify the sources of heterogeneity, also we drew a Random Gal braith plot for PFS (Fig. 3b), suggesting that the studies conducted by YANG CHEN [38], XIUMEI Z [40], and SATOSHI [81] could be used as sources of heterogeneity.

### Subgroup analyses for incidence of CTCs and prognosis of overall survival

For the subgroup analysis, we needed to categorize the obtained data. The detection of markers in the included studies was very different; therefore, we divided it into terms of both epithelial markers and mesenchymal markers. Also, all of the studies were classified into two-stage groups (clinical stage and TNM stage) if provided. Detection methods for all 45 studies applied to cell search method and other methods for comparison.

In OS, we had a low heterogeneity, but for the assessment of changes in HR results observed in different groups, we performed a subgroup analysis for markers, detection methods, treatment type, presence of distance metastasis, presence of lymph node metastasis, and risk of bias in the overall estimates of meta-analysis on the correlation between the incidence of CTCs and the prognosis of overall survival (Table 2). In all groups, results were statistically significant, and *P* value for heterogeneity between subgroups was not statistically significant.

**Table 2:** Overall estimates of meta-analysis conducted on the correlation between incidence rate of CTCs and the prognosis of overall survival of PFS

Outcomes	Subgroups	*N*	HR (95% CI)	*P* value	*I*^2^ (%)	*P* heterogeneity	*P* heterogeneity between subgroups	References
OS		21	2.75 (2.34, 3.24)	< 0.001	22.8	0.169	–	[37, 38, 40–42, 44, 47, 48, 54, 57, 61–63, 66, 67, 71, 72, 75, 79, 81]
Marker	Epithelial	15	2.87 (2.38, 3.46)	< 0.001	38.8	0.063	0.870	[38, 40, 41, 44, 48, 56, 57, 61–63, 71, 72, 75, 79, 81]
	Mesenchymal	3	2.30 (1.50, 3.53)	< 0.001	0.0	0.793		[42, 47, 50]
	Epithelial + Mesenchymal	2	2.54 (1.45, 4.45)	0.001	38.0	0.204		[37, 66]
	Not reported	1	2.28 (0.25, 20.15)	0.459	–	–		[80]
Detection methods	Cell search	9	2.61 (2.05, 3.32)	< 0.001	49.7	0.044	0.560	[41, 44, 48, 56, 61, 71] [72, 79, 81]
	Cytological	12	2.87 (2.31, 3.58)	< 0.001	0.0	0.560		[37, 38, 40, 42, 47, 50] [57, 62, 63, 66, 67, 75]
Treatment type	Surgery	5	3.06 (2.04, 4.61)	< 0.001	57.1	0.054	0.739	[50, 56, 57, 67, 79]
	Chemotherapy	15	2.68 (2.23, 3.20)	< 0.001	12.4	0.314		[37, 38, 40–42, 44, 47, 48, 61, 62, 66, 71, 72, 75, 81]
	No treatment	1	3.70 (1.20, 12.40)	0.028	–	–		[63]
Presence of distance metastasis	Yes	17	2.67 (2.24, 3.18)	< 0.001	20.3	0.216	0.336	[37, 38, 40–42, 44, 47, 48, 57, 61, 66, 67, 71, 72, 75, 79, 81]
	No	4	3.39 (2.15, 5.35)	< 0.001	38.8	0.179		[50, 56, 62, 63]
Presence of lymph node metastasis	Yes	10	2.93 (2.12, 4.05)	< 0.001	48.5	0.042	0.886	[38, 40, 41, 48, 57, 67, 72, 79, 81]
	No	11	2.79 (2.18, 3.57)	< 0.001	0.0	0.587		[37, 42, 44, 47, 50, 56, 61–63, 66, 71]
Risk of bias	Low	14	3.08 (2.49, 3.81)	< 0.001	35.3	0.093	0.114	[40, 42, 44, 47, 48, 50, 56, 57, 61, 62, 66, 72, 75, 81]
	High	7	2.36 (1.84, 3.03)	< 0.001	0.0	0.767		[37, 38, 41, 63, 67, 71, 81]
PFS		17	2.78 (2.01, 3.85)	< 0.001	70.9	< 0.001	–	[37–41, 48, 50, 56, 61, 62, 66, 67, 71, 72, 75, 79, 81]
Marker	Epithelial	13	2.94 (2.01, 4.29)	< 0.001	75.7	< 0.001	0.910	[38–41, 48, 56, 61, 62, 71, 72, 75, 79, 81]
	Mesenchymal	1	2.36 (1.03, 5.37)	0.041	–	–		[50]
	Epithelial + Mesenchymal	2	3.03 (0.47, 19.44)	0.242	74.3	0.048		[37, 66]
	Not reported	1	2.06 (0.72, 5.97)	0.179	–	–		[67]
Detection	Cell search	8	3.25 (2.14, 4.93)	< 0.001	68.1	0.003	0.331	[41, 48, 56, 61, 71, 72] [79, 81]
	Other	9	2.32 (1.37, 3.95)	0.002	74.8	< 0.001		[37–40, 50, 62, 66, 67, 75]
Treatment type	Surgery	5	2.07 (1.45, 2.96)	< 0.001	49.4	0.095	0.287	[39, 50, 56, 67, 79]
	Chemotherapy	12	3.01 (2.03, 4.46)	< 0.001	76.1	< 0.001		[37, 38, 40, 41, 48, 61, 62, 66, 71, 72, 75, 81]
Presence of distance metastasis	Yes	13	2.80 (1.94, 4.03)	< 0.001	75.2	< 0.001	0.525	[37, 38, 40, 41, 48, 61, 66, 67, 71, 72, 75, 79, 81]
	No	4	2.86 (1.76, 4.67)	< 0.001	52.3	0.099		[39, 50, 56, 62]
Presence of lymph node metastasis	Yes	9	2.66 (1.70, 4.16)	< 0.001	79.0	< 0.001	0.715	[38, 40, 41, 48, 67, 72, 75, 79, 81]
	No	8	3.00 (1.87, 4.81)	< 0.001	54.1	0.033		[37, 39, 50, 56, 61, 62, 66, 71]
Risk of bias	Low	11	3.11 (2.22, 4.34)	< 0.001	50.7	0.027	0.452	[39, 40, 48, 50, 56, 61, 62, 66, 72, 75, 79]
	High	6	2.37 (1.28, 4.40)	0.006	81.7	< 0.001		[37, 38, 41, 67, 71, 81]

N: Number of studies, RD: Risk Difference

In PFS, we had a high heterogeneity and for the detection of sources of heterogeneity, we needed to run a subgroup analysis for markers, detection methods, treatment type, presence of distance metastasis, presence of lymph node metastasis, and risk of bias. The results showed that treatment type and absence of lymph node metastasis might be considered as sources of heterogeneity (Table 2).

### Overall estimates of meta-analysis on the risk differences (RDs) of the presence of CTCs in sampling time

The incidence rate of CTCs was assessed based on the sampling time points reported in the 13 studies at baseline (before surgery or chemotherapies) and after the treatment (during or after operations and chemotherapies) (Supplementary Table 2). RD analysis results demonstrated that intervention could decrease incidence rate of CTCs (RD: − 0.17, 95%CI (-0.28, − 0.06), *P* 0.002), indicating a high heterogeneity (*I*^2^ 89.0%, *p* =  < 0.001) (Supplementary Fig. 2). Sensitivity analysis results did not show further information. Visual inspection of funnel plot, the Begg’s test (*P* = 0.542) and Egger’s test (*P* = 0.464) showed no publication bias. Subgroups analysis was performed to identify sources of heterogeneity, and the *P* value of heterogeneity between subgroups was not statistically significant.

RD analysis results showed that the incidence rate of CTCs for mesenchymal marker (RD: − 0.35, 95%CI (-0.57, − 0.13), *p* 0.002), epithelial marker (RD: − 0.12, 95%CI (-0.25, 0.00), *p* 0.05), cell search method (RD: − 0.19, 95%CI (-0.28, − 0.10), *p* < 0.001), chemotherapy treatment (RD: − 0.17, 95%CI (-0.31, − 0.03), *p* 0.016), presence of distance metastasis (RD: − 0.21, 95%CI (-0.34, − 0.07), *p* 0.002) and absence of lymph node metastasis (RD: − 0.18, 95%CI (-0.29, − 0.06), *p* 0.002)) decreased after the treatment as compared with before the treatment.

### Overall estimates of meta-analysis on the risk differences (RD) of the presence of CTCs in various stages

Of the 45 studies reviewed, 21 reported incidence rates of CTC in the clinical stage (I-IV), 11 reported it in the TNM stage and 4 studies reported it in both stages (53.63.86.195). For RD analysis of the presence of CTCs in the clinical stage, we categorized it into the first clinical stage and TNM stage that in clinical stage we had both the early stage (I and II) and the advanced stage (III and IV). Also, in the TNM stage, we categorized it into intestinal, diffuse and mix and assessed the risk of CTCs per group (Table 3).

**Table 3:** Overall estimates of meta-analysis on the risk differences (RDs) of the presence of CTCs in various stages

Outcomes	Subgroups	*N*	RD (95% CI)	*P* value	*I*^2^ (%)	*P* heterogeneity	*P* heterogeneity between subgroups
Stage: early vs. advanced		21	− 0.10 (− 0.23, 0.02)	0.105	87.0	< 0.001	–
Marker	Epithelial	12	− 0.12 (− 0.29, 0.05)	0.162	89.2	< 0.001	0.605
	Mesenchymal	3	− 0.22 (− 0.36, − 0.09)	0.001	39.9	0.189	
	Epithelial + Mesenchymal	4	− 0.01 (− 0.33, 0.30)	0.940	87.9	< 0.001	
	Not reported	2	0.01 (− 0.56, 0.58)	0.969	85.1	0.010	
Detection	Cell search	4	− 0.38 (− 0.64, − 0.12)	0.004	91.5	< 0.001	0.011
	Other	17	− 0.02 (− 0.23, 0.02)	0.761	74.7	< 0.001	
Treatment type	Surgery	11	− 0.08 (− 0.24, 0,08)	0.32	86.3	< 0.001	0.38
	Chemotherapy	8	− 0.18 (− 0.41, 0.04)	0.11	86.5	< 0.001	
	No Treatment	2	0.11 (− 0.22, 0.02)	0.53	80.9	< 0.001	
Presence of distance metastasis	Yes	15	− 0.16 (− 0.29, − 0.02)	0.020	83.5	< 0.001	0.267
	No	6	0.03 (− 0.27, 0.33)	0.851	91.8	< 0.001	
Presence of lymph node metastasis	Yes	13	− 0.09 (− 0.25, 0.06)	0.238	89.1	< 0.001	0.863
	No	8	− 0.12 (− 0.33, 0.10)	0.281	81.2	< 0.001	
Risk of bias	Low	12	− 0.21 (− 0.37, − 0.05)	0.012	90.1	< 0.001	0.011
	High	9	0.07 (− 0.07, 0.21)	0.328	59.1	0.012	
Stage: intestinal vs. diffuse		11	− 0.19 (− 0.37, − 0.01)	0.045	85.7	< 0.001	–
Marker	Epithelial	6	− 0.23 (− 0.44, − 0.01)	0.040	85.2	< 0.001	0.443
	Mesenchymal	1	− 0.43 (− 0.74, − 0.12)	0.006	–	–	
	Epithelial + Mesenchymal	3	− 0.04 (− 0.46, 0.37)	0.845	85.5	0.001	
	Not reported	1	− 0.15 (− 0.45, 0.15)	0.340	–	–	
Detection	Cell search	4	− 0.33 (− 0.57, − 0.10)	0.006	85.4	< 0.001	0.135
	Other	7	− 0.09 (− 0.31, 0.14)	0.450	78.6	< 0.001	
Treatment type	Surgery	3	− 0.19 (− 0.70, 0.32)	0.47	91.8	< 0.001	0.98
	Chemotherapy	8	− 0.18 (− 0.37, 0.01)	0.06	83.1	< 0.001	
Presence of distance metastasis	Yes	9	− 0.18 (− 0.35, − 0.001)	0.048	80.7	< 0.001	0.952
	No	2	− 0.20 (− 1.00, 0.61)	0.626	95.7	< 0.001	
Presence of lymph node metastasis	Yes	3	− 0.19 (− 0.30, − 0.07)	0.002	0.0	0.944	0.793
	No	8	− 0.17 (− 0.25, − 0.09)	< 0.001	90.0	< 0.001	
Risk of bias	Low	7	− 0.28 (− 0.50, − 0.06)	0.014	83.8	< 0.001	0.086
	High	4	− 0.02 (− 0.22, 0.18)	0.865	68.2	0.024	
Stage: intestinal vs. mixed		8	− 0.11 (− 0.34, 0.12)	0.348	88.1	< 0.001	–
Marker	Epithelial	5	− 0.10 (− 0.43, 0.23)	0.561	90.5	< 0.001	0.928
	Epithelial + Mesenchymal	3	− 0.12 (− 0.45, 0.21)	0.472	82.0	0.004	
Detection	Cell search	3	− 0.23 (− 0.67, 0.21)	0.311	92.6	< 0.001	0.433
	Other	5	− 0.03 (− 0.26, 0.20)	0.796	75.8	0.002	
Treatment type	Surgery	2	− 0.15 (− 1.11, 0.80)	0.756	96.4	< 0.001	0.86
	Chemotherapy	6	− 0.06 (− 0.21, 0.08)	0.390	61.1	0.025	
Presence of distance metastasis	Yes	6	− 0.07 (− 0.22, 0.08)	0.390	61.1	0.025	0.862
	No	2	− 0.15 (− 1.11, 0.81)	0.756	96.4	< 0.001	
Presence of lymph node metastasis	Yes	1	− 0.11 (− 0.34, 0.12)	0.352	–	–	0.991
	No	7	− 0.11 (− 0.38, 0.16)	0.415	89.8	< 0.001	
Risk of bias	Low	5	− 0.17 (− 0.54, 0.21)	0.381	91.3	< 0.001	0.003
	High	3	− 0.005 (− 0.12, 0.11)	0.937	51.5	0.127	
Stage: diffuse vs. mixed		8	0.05 (− 0.12, 0.22)	0.534	76.0	< 0.001	–
Marker	Epithelial	5	0.14 (− 0.11, 0.38)	0.287	83.8	< 0.001	0.027
	Epithelial + Mesenchymal	3	− 0.08 (− 0.22, 0.06)	0.260	0.0	0.830	
Detection	Cell search	3	0.16 (− 0.22, 0.53)	0.407	91.6	< 0.001	0.069
	Other	5	− 0.03 (− 0.16, 0.09)	0.585	0.0	0.726	
Treatment type	Surgery	2	− 0.04 (− 0.13, 0.12)	0.957	0.0	0.39	0.286
	Chemotherapy	6	0.05 (− 0.18, 0.29)	0.641	81.7	< 0.001	
Presence of distance metastasis	Yes	6	0.06 (− 0.18, 0.30)	0.641	81.7	< 0.001	0.286
	No	2	− 0.004 (− 0.13, 0.13)	0.957	0.0	0.396	
Presence of lymph node metastasis	Yes	1	0.06 (− 0.27, 0.39)	0.717	–	–	0.971
	No	7	0.05 (− 0.14, 0.24)	0.583	79.4	< 0.001	
Risk of bias	Low	5	0.10 (− 0.17, 0.37)	0.462	84.2	< 0.001	0.072
	High	3	− 0.05 (− 0.18, 0.09)	0.496	0.0	0.766	

The RDs in the clinical early and advanced stages were not statistically significant, but RD value was negative (RD: − 0.10, 95%CI (-0.23, 0.02), *P* 0.105). Heterogeneity rate was high (*I*^2^ 87.0%, *p* =  < 0.001). Subgroup analysis was performed for markers, detection methods, treatment type, presence of distance metastasis, presence of lymph node metastasis, and risk of bias and RD values for mesenchymal markers (RD: − 0.22, 95%CI (-0.36, − 0.09), *P* 0.001), cell search (RD: − 0.38, 95%CI (-0.64, − 0.12), P0.004) and low risk of bias (RD: − 0.21, 95%CI (-0.37, − 0.05), *P* 0.012) were statistically significant. *P*-values of heterogeneity between subgroups in the detection methods (*P*: 0.011) and risk of bias (*P*: 0.011) were statistically significant, showing that detection methods and risk of bias could be considered as sources of heterogeneity. Sensitivity analysis did not show further information. Visual inspection of the funnel plot and the Begg’s test (*P* = 0.809) and Egger’s test (*P* = 0.998) did not show publication bias.

The RDs in the TNM stage between intestinal vs diffuse, intestinal vs mixed and diffuse vs mixed were analyzed (Table 3). RD analysis in intestinal type vs diffuse type showed that the incidence rate of CTCs in the diffuse stage was higher than that of intestinal stage (RD: − 0.19, 95%CI (-0.37, − 0.01), P0.045), indicating a high heterogeneity (*I*^2^ 85.7%, *p* =  < 0.001). Also, epithelial markers (RD: − 0.23, 95%CI (-0.44, − 0.01), *p* 0.040), mesenchymal markers (RD: − 0.43, 95%CI (-0.74, − 0.12), *p* 0.006), cell search method (RD: − 0.33, 95%CI (-0.57, − 0.10), *p* 0.006), presence of distance metastasis (RD: − 0.18, 95%CI (-0.35, − 0.001), *P* 0.048), presence of lymph node (RD: − 0.19, 95%CI (-0.30, − 0.07), *p* 0.002) and studies with a low risk of bias (RD: − 0.28, 95%CI (-0.50, − 0.06), *p* 0.014) were significant that the use of these variables indicated the detection risk of CTCs in diffuse type was greater than that in intestinal one. the Sensitivity analysis results demonstrated that no significant difference was observed between intestinal type and diffuse type in terms of the risk of CTC presence after removing the studies conducted by Q. Zhang (2018) (RD = -0.14, 95% CI = -0.31, 0.03), B Cheng (2019) (RD = -0.16, 95% CI = -0.35, 0.03), Simon Pernot (2017) (RD = -0.15, 95% CI = -0.34, 0.03), Joon Hyung Jhi (2021) (RD = -0.16, 95% CI = -0.35, 0.03), H. Okabe (2015) (RD = -0.18, 95% CI = -0.40, 0.03), Yang Chen (2021) (RD = -0.19, 95% CI = -0.38, 0.01), L. Zheng (2017) (RD = -0.19, 95% CI = -0.38, 0.01), and Yilin Li (2015) (RD = -0.20, 95% CI = -0.40, 0.001) (Supplementary Fig. 3). Visual inspection of funnel plot, the Begg’s test (*P* = 0.938) and Egger’s test (*P* = 0.803) showed no publication bias.

RD analysis results in the intestinal type vs mixed type were not statistically significant (RD: − 0.11, 95% (-0.34, 0.12), *p* 0.348), indicating a high heterogeneity (*I*^2^ 88.1%, *p* =  < 0.001). * P* values of heterogeneity between subgroups in the studies with a low risk of bias (*p* 0/003), which could be a source of heterogeneity, were statistically significant. Sensitivity analysis did not show further information. Visual inspection of funnel plot, the Begg’s test (*P* = 1.000) and Egger’s test (*P* = 0.541) showed no publication bias (Table 3).

Also, RD analysis results obtained for the diffuse type vs. mixed type (RD: 0.05, 95% (-0.12, 0.22), *P* 0.534) showed that the incidence rate of CTCs in diffuse type was more than that in mix type. Sensitivity analysis did not show further information. Visual inspection of funnel plot, the Begg’s test (*P* = 0.322) and Egger’s test (*P* = 0.958) showed no publication bias (Table 3).

### Meta-regression analysis

Meta-regression analysis was performed to compare incidence rate of CTCs with OS, PFS and RDs. Also, meta-regression was employed to examine the effect of baseline characteristics (such as age, male-to-female ratio, follow-up duration, distance metastasis and lymph node metastasis). The results of meta-regression were not statistically significant, and all of the analysis in meta-regression did not show further information (Supplementary Table 3).

## Discussion

In this study, we provided the most comprehensive systematic review and meta-analysis performed on role of CTCs in GC. Also, many variables used for the role of CTCs after using many advanced statistical methods in this meta-analysis were described. These methods contribute to get a deeper and more comprehensive result after adjusting for clinical factors; meta-analysis offers compelling evidence. Novel findings are obtained from our meta-analysis. Also, our results are significantly larger than those of previous studies conducted on CTCs consisting of 3342 GC patients, and making it the most thorough systematic assessment showing the correlation between CTCs and GC prognosis to date [20–26].

Despite the progress in the diagnosis and management of cancer, a significant reduction in GC mortality was still not achieved. The value of CTCs evaluation in cancer management was approved by previous studies. However, in the present meta-analysis, we investigated the best interpretations regarding the use of CTCs to improve GC survival so that their applications can be validated for treatment strategies.

In the previous meta-analyses [20–26], there was an association between poor PFS and OS in GC patients, but most of them were no considered as the comprehensive meta-analyses. In this meta-analysis, we provided strong evidence, suggesting that a significant association was observed between poor PFS and OS in GC patients, irrespective of the geographical location, population, age, gender, tumor stage, TNM stage, maker studied, detection methods, CTC incidence, follow-up duration, type of clinical therapy, blood sample volume, timing of blood sample collection, distance and lymph node metastasis.

Except for the study of Yunhe Gao et al. [24], for studies where HRs were not provided no statistical analysis was performed to calculate the approximated HRs. In addition to estimating HRs for some studies [37–42], we reported a significant publication bias (*P*-value Begg’s test = 0.022, *P*-value for Egger’s test = 0.025), which the HR (95% CI) for OS was changed to 2.37 (2.04, 2.74) after the imputation of 6 studies in the nonparametric trim-and-fill test. Also, in the HR for PFS (HR = 2.78, 95%CI 2.01–3.85, *p* < 0.001), we showed that the studies conducted by YANG CHEN [38], XIUMEI Z [40], and SATOSHI [81] can be considered as sources of heterogeneity. Moreover, in PFS, as epithelial and mesenchymal markers (HR: 3.03, 95%CI (0.47, 19.44), cell search methods (HR: 3.25, 95%CI (2.14, 4.93), chemotherapy (HR: 3.01, 95%CI (2.03, 4.46), absence of lymph node metastasis (HR: 3.00, 95%CI (1.87, 4.81) and low-risk bias (HR: 3.11, 95%CI (2.22, 4.34) were observed, progression or recurrence of GC in patients who had CTC positivity prognosis was increased. Therefore, our results with high accuracy showed that CTCs observed in the peripheral blood was predictive of a poorer survival outcome.

Subgroup analyses of OS and PFS were performed based on markers, detection techniques, treatment type, presence of distance metastasis, presence of lymph node metastasis, and risk of bias in the overall estimates of meta-analysis performed on the correlation between the incidence rate of CTCs and prognosis of overall survival in all groups, indicating significant results. Also, *P* value of heterogeneity between subgroups was not statistically significant. The results obtained from PFS showed that treatment type and absence of lymph node metastasis could be considered as sources of heterogeneity. The results of the subgroup analyses were consistent with those of overall analyses, but this coordination was not seen in some previous meta-analyses [21–23, 25], because there are some limitation for them so that heterogeneity was considered as the greatest problem in these subgroup analyses [23, 25] or a few variable were analyzed and small studies were included in their meta-analyses [23–25], Therefore, the results of the present study were more reliable than those of the previous study, but both studies achieved the same conclusion.

To date, the cell search system can be regarded as the only US FDA-certified CTC enumeration assay, which can define CTCs based on their size, positivity for epithelial cell adhesion molecule (EPCAM) and cytokeratin, and negative cluster of differentiation 45 (CD45) antigen expressions, but there is be a controversy in studies regarding cell search and other methods because CTCs are generally assumed to be extremely heterogeneous in both phenotype and genotype. Some specific CTCs may be overlooked in some techniques; for example, the CTCs undergoing the epithelial-to-mesenchymal transition (EMT) could hardly be detected by using cell search method and may be identified by using other approaches. The results of this meta-analysis solved this controversy and also subgroup analysis results showed that the prognostic role of CTCs which was detected by Cell Search and cytological techniques (such as MACS, FACS, FACS-ICC, SE‑iFISH, Im-FISH, CanPatrol CTC enrichment, Immune-magnetic, immunocytochemistry, Meta Cell) was significant in both methods. Also, markers used in this method were divided into epithelial markers or mesenchymal markers or both of them (epithelial + mesenchymal) that the significant were according to our results all markers. Therefore, it seems that detection methods whether based on epithelial markers or mesenchymal marker are appropriate for a sample of CTCs undergoing EMT in GC.

In their meta-analysis, Hui-Yu Wang et al. [25] compared the CTC detection methods, and the results demonstrated that the prognostic value was not statistically significant. In their study, Kun Zou et al. [23] also showed that cell search methods were non-significant. Shuyi Wang et al. [25] demonstrated that RT-PCR was more sensitive than other methods used for the detection of CTCs, but there are some limitation ( small study include for analysis and heterogeneity in this studies) can be the reason for the difference between our results and theirs. Also, we found that the data were more finely as compared with the previous study, However, the results of the studies conducted by Yunhe Gao et al. [24] and Chaogang Yang [26] were consistent with our study; however, we believe that the new detection techniques would continuously appear and should be considered for future studies [82].

Another important and debatable variable in past studies is the type of therapy used for GC patients, as the results obtained from our meta-analysis demonstrated that patients in surgery group and chemotherapy group reported a significant prognostic value, but surgery group with the presence of CTCs in blood samples showed shorter survival time. In other words, if CTCs were detected in blood samples collected during surgery, they showed a poorer OS that might be compatible with Hou JM’s point of view that CTCs can promote the metastasis [83].

One of the most important features of this study was the investigation of the RD. This was the first meta-analysis that examined the effect of the presence of CTCs before and after the intervention.

Regarding the division of the included studies based on sampling time, our results demonstrated that the use of the cell search method and epithelial or mesenchymal markers before treatment can have a higher diagnostic power to identify the CTCs.

Recently, the evaluation of CTC kinetics has received increasing attention. Pachmann et al. [84] also found that in breast cancer there were many relapses in the groups in which the number of CTCs enhanced. Furthermore, Li et al. [73] reported that the patients with worsened medical conditions showed a high rate of mesenchymal CTCs. Moreover, Ishiguro et al. [46] showed that CTCs alive should be evaluated to predict the metastasis. Also, they found that the approximate estimation of estimating the time of recurrence is possible by dividing patients according to the presence of CTCs before and after the treatment. In this meta-analysis, we found that a high status of CTCs after chemotherapy could significantly reduce the number of CTCs, mesenchymal markers, and presence of distance metastasis which has a high status before the treatment; therefore, this theory that is recently proposed [23] that “CTCs can be removed by chemotherapeutic medicines via both the direct and indirect mechanisms, including cytotoxic and anti-metabolic impacts and the remaining CTCs after the chemotherapy may be more aggressive than before, and it may be easy to create metastases or lead to recurrence” which is consistent with our study. It should be kept in mind that heterogeneity could be considered as the greatest problem in these subgroup analyses. Therefore, further studies with sufficient key data are needed to get further understanding of the detection of the CTCs in GC patients at different time points.

We also surveyed RDs in clinical stage in GC as early stage (I-II) and advance stage (III-IV). For this purpose, we determined the risk of CTCs in the early and advanced stages and calculated their difference. As expected, the RDs in the clinical early and advanced stages were not statistically significant (RD: − 0.10, 95%CI (-0.23, 0.02), *P* 0.105. The results of these analyses demonstrated that the mesenchymal markers and the cell search method have a higher power to identify CTCs in the advanced stage than in the early stage. In our opinion, if cytological methods are used to isolate CTC in GC patients, it is better to use mesenchymal markers when a patient is in advanced stage because epithelial markers may be ignored in a population of CTCs that have undergone EMT. Therefore, the use of mesenchymal markers (such as N-cadherin,…) for the cellular heterogeneity of CTCs is useful in these conditions [46], although the cell search method still proves acceptable results.

Our investigations about the stage did not end only in the clinical stage. Regarding the TNM classification, the results showed that the incidence rate of CTCs in the diffuse type was higher than that in the intestinal type, and also both mesenchymal and epithelial markers have a higher power to isolate CTC in addition to the cell search methods. Our results were in agreement with Hui-Yu Wang's [25] meta-analysis. According to the results of this study, it seems that CTCs could present useful information for both tumor staging and the diagnosis of cancer [85].

We observed no significant difference between mix type and intestinal type in terms of the incidence rate of CTCs, but we found that the chance of CTCs in diffuse type was higher than that in mix type. However, the results of this section should be interpreted with caution because of limited studied (*n* = 8).

Finally, meta-regression analysis results showed that baseline characteristics (such as age, male to female ratio, follow-up duration, distance metastasis and lymph node metastasis) were not correlated with the detection of CTCs in GC patients.

### Future directions

In this study, we tried to examine many variables contributing to role of CTCs in GC and respond to the controversy topics in different studies, but still there are many things that are a future perspective, firstly, concerning CTCs, the main issue is their survival than circulation because it was understood that in addition to metastasis, CTCs could return to the primary tumor and causes more tumor aggravation [86]. Therefore, it can be concluded that the absence of CTCs does not necessarily indicate a good prognosis. Secondly, the primary gene abnormality influences the CTCs' behaviors. Gkountela et al.'s [87] study showed that the hypermethylation in primary involved gene causes clustering CTCs accompanied by poor prognosis. Thirdly, the source of the CTC samples gives different information. In a study conducted by Liu et al., CTCs of arterial blood are more valuable than venous blood. Regarding the phenotype of CTCs, they concluded that low epithelial cells have better outcomes [88]; however, fewer cells in arterial blood are more favorable prognoses. Additionally, their study demonstrated that arterial blood is recommended for evaluating gene mutations in CTCs [88]. A study that aimed to determine the role of blood flow in cancer metastasis showed that, on average, 40% of cancer metastasis is influenced by blood flow [89]. Fourthly, transcriptome analysis of single CTCs has only been reported for a limited number of cancer types. Recently, Negishi et al. [90] found that the transcriptome analysis of gastric cancer single CTCs revealed that platelet adhesion could contribute to EMT progression and acquisition of chemoresistance. However, more studies are needed to employ CTC characterization in order to elucidate the mechanisms of chemoresistance and metastasis in GC.

### Limitations

We acknowledge some limitations of the large-scale meta-analysis. First, we tried to minimize the publication bias with various statistical tests, but some studies might tend to selectively present their positive findings, resulting in risk of both the selection and publication bias. Second, the majority of studies were limited to eastern Asia because of the low morbidity rates observed in western countries, which may influence the external validity of these findings for GC globally. Third, we searched for studies without the limitation of time, but we did not search for unpublished data. Therefore, some missing and unpublished data may not be included in current study, which may influence the pooled results. Forth, the meta-analysis employed the pooled data extracted from heterogeneous studies, but not original data obtained from each patient. Furthermore, several studies did not present HRs and thus the reported data were used to estimate them. Fifth, multiple methods for CTCs detection were used in our meta-analysis, that was not possible to compare two by two to determine the strength of each of the methods. Sixth, heterogeneity was observed between studies due to several detection methods, different cutoff values of CTCs, etc. We tried to solve this problem by extracting more data from studies and performing subgroup analyses. However, a significant heterogeneity was observed in some subgroups and also a random-effects model was used for more conservative estimations. Hence, it is therefore recommended that large multicenter prospective studies enrolling homogeneous populations to be conducted to validate the prognostic value of CTC detection and more accuracy risk difference. Seventh, the current meta-analysis is the lake of data on inflammatory biomarkers. Inflammation cytokines have a pivotal role in creating and promoting the situations of tumor aggression. In this regard, it is highly recommended that future studies to be investigated the relationship between inflammation cytokines and the formation of CTCs.

## Conclusion

According to our systematic review and meta-analysis, evidence provided the significant prognostic value of CTCs detected for both PFS and OS in GC patients, and the detection of CTCs was correlated some clinic-pathological features. Overall prevalence (%) of CTCs in GC was 69.37 (60.27, 77.78), and also risk of CTC in the advanced stage was higher than that in the early stage. The results show that finding CTCs can be an ideal technique for enhancing the prognosis of patients with gastric cancer and customized patient follow-up. The result of prognostic role of CTCs detected by Cell Search and cytological methods showed that detection methods whether based on epithelial markers or mesenchymal markers may be appropriate for a population of CTCs in GC. Risk difference analysis results showed which intervention could decrease incidence rate of CTCs so that chemotherapy could significantly reduce the number of CTCs. Also, mesenchymal markers and presence of distance metastasis have a high status before the treatment. CTCs were detected in blood samples collected during surgery, indicating a poorer OS. The outcomes of CTC detection may also be utilized in the future to create personalized medicine programs. However, CTCs identification may be suggested as a diagnostic technique for gastric cancer screening, but the findings must be interpreted with caution. It is therefore recommended that large-scale prospective studies on multiple regions be conducted to obtain more accurate data.

## Supplementary Information

Below is the link to the electronic supplementary material.

Supplementary file1 (DOCX 201 KB)

Supplementary file2 (DOCX 1561 KB)

Supplementary file3 (DOCX 1732 KB)

Supplementary file4 (DOCX 43 KB)

Supplementary file5 (DOCX 51 KB)

Supplementary file6 (DOCX 15 KB)
