# Supplementary figures and images for: Investigating the role of circulating tumor cells in gastric cancer: a comprehensive systematic review and meta-analysis
Source: Clin Exp Med. 2024 Mar 30;24(1):59. doi: 10.1007/s10238-024-01310-6 (PMC10981629; doi:10.1007/s10238-024-01310-6)

Supplementary Figure 1. Risk of bias assessments


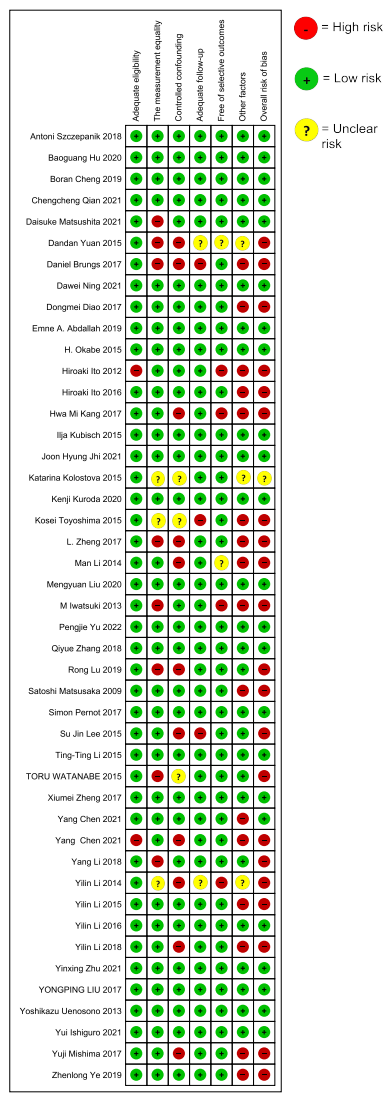


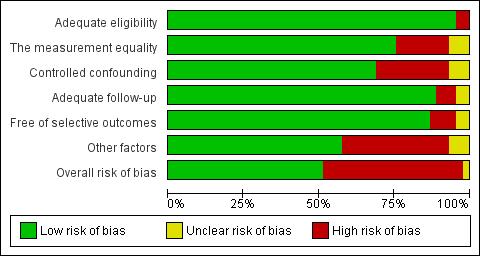

Supplement: Supplementary file 1 — Supplementary file1 (DOCX 201 KB) [file 10238_2024_1310_MOESM1_ESM.docx]

Supplementary Figure 2. Risk differences (RD) of the presence of CTCs in sampling time


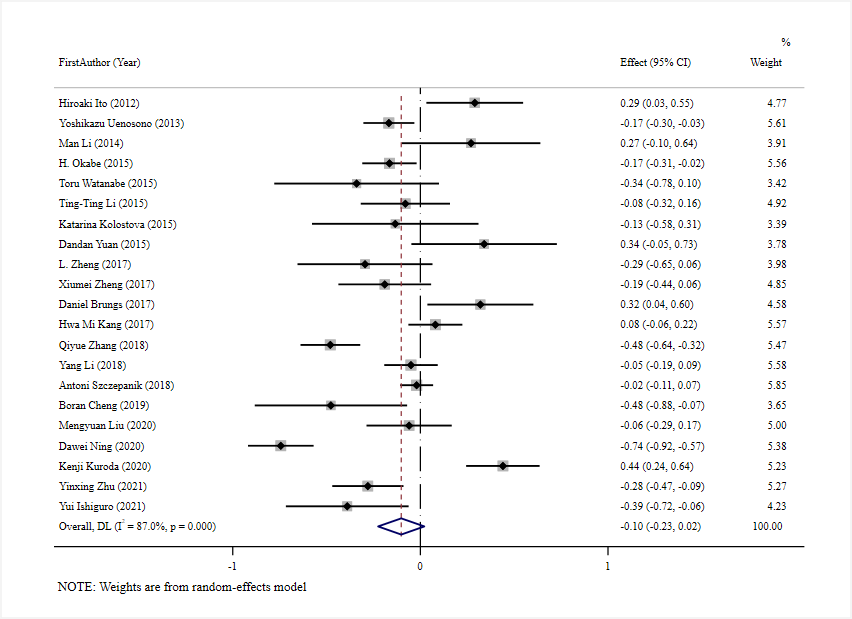

Supplement: Supplementary file 2 — Supplementary file2 (DOCX 1561 KB) [file 10238_2024_1310_MOESM2_ESM.docx]

Supplementary Figure 3. Sensitivity analysis performed in intestinal and diffuse stages


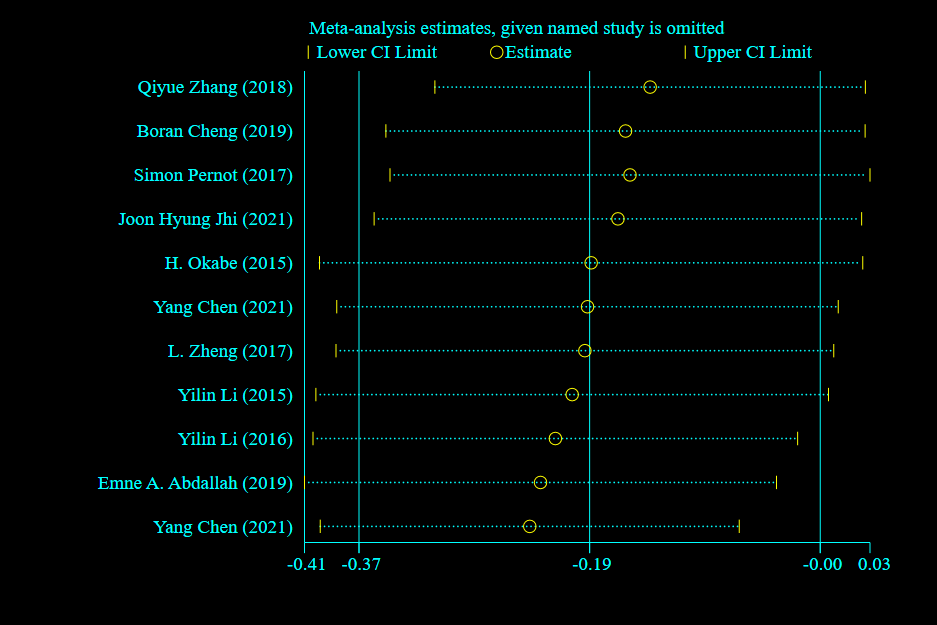

Supplement: Supplementary file 3 — Supplementary file3 (DOCX 1732 KB) [file 10238_2024_1310_MOESM3_ESM.docx]
