# Supplementary material for: Investigating the role of circulating tumor cells in gastric cancer: a comprehensive systematic review and meta-analysis
Source: Clin Exp Med. 2024 Mar 30;24(1):59. doi: 10.1007/s10238-024-01310-6 (PMC10981629; doi:10.1007/s10238-024-01310-6)
Supplement: Supplementary file 4 — Supplementary file4 (DOCX 43 KB) [file 10238_2024_1310_MOESM4_ESM.docx]

Supplementary Table 1. Cochrane Risk of study.

| Study | Q1 | Q2 | Q3 | Q4 | Q5 | Q6 | Quality Score | Risk of bias? |
| --- | --- | --- | --- | --- | --- | --- | --- | --- |
| Yang Chen  (Chen, Li et al. 2021) | Yes | Yes | Yes | Yes | Yes | No | 5/6 | High |
| Yang Chen  (Chen, Yuan et al. 2021) | No | Yes | No | Yes | Yes | No | 3/6 | High |
| Chengcheng Qian  (Qian, Cai et al. 2021) | Yes | Yes | Yes | Yes | Yes | Yes | 6/6 | Low |
| Daisuke Matsushita (Matsushita, Uenosono et al. 2021) | Yes | No | Yes | Yes | Yes | Yes | 5/6 | High |
| Yui Ishiguro  (Ishiguro, Sakihama et al. 2021) | Yes | Yes | Yes | Yes | Yes | Yes | 6/6 | Low |
| Yinxing Zhu  (Zhu, Chen et al. 2021) | Yes | Yes | Yes | Yes | Yes | Yes | 6/6 | Low |
| Dawei Ning  (Ning, Cui et al. 2021) | Yes | Yes | Yes | Yes | Yes | Yes | 6/6 | Low |
| Kenji Kuroda  (Kuroda, Yashiro et al. 2020) | Yes | Yes | Yes | Yes | Yes | Yes | 6/6 | Low |
| Mengyuan Liu  (Liu, Wang et al. 2020) | Yes | Yes | Yes | Yes | Yes | Yes | 6/6 | Low |
| E A. Abdallah (Abdallah, Braun et al. 2019) | Yes | Yes | Yes | Yes | Yes | Yes | 6/6 | Low |
| Antoni Szczepanik (Szczepanik, Sierzega et al. 2019) | Yes | Yes | Yes | Yes | Yes | Yes | 6/6 | Low |
| Boran Cheng (Cheng, Tong et al. 2019) | Yes | No | Yes | No | Yes | Yes | 4/6 | High |
| Rong Lu (Lu, Chen et al. 2019) | Yes | No | No | Yes | Yes | Yes | 4/6 | High |
| Study | **Q1** | **Q2** | **Q3** | **Q4** | **Q5** | **Q6** | **Quality Score** | **Risk of bias?** |
| Yang Li(Li, Ma et al. 2018) | Yes | Yes | No | Yes | Yes | No | 4/6 | High |
| Qiyue Zhang (Zhang, Shan et al. 2018) | Yes | Yes | Yes | Yes | Yes | Yes | 6/6 | Low |
| Ting-Ting Li (Li, Liu et al. 2015) | Yes | No | Unclear | Yes | Yes | Unclear | 3/4 | High |
| Yuji Mishima (Mishima, Matsusaka et al. 2017) | Yes | Yes | No | Yes | Yes | No | 4/6 | High |
| Diao  Dongmei (Diao, Cheng et al. 2017) | Yes | Yes | Yes | Yes | Yes | Yes | 6/6 | Low |
| Xiumei Zheng (Zheng, Fan et al. 2017) | Yes | Yes | Yes | Yes | Yes | Yes | 6/6 | Low |
| Simon Pernot (Pernot, Badoual et al. 2017) | Yes | Yes | Yes | Yes | Yes | Yes | 6/6 | Low |
| Toru Watanabe (Watanabe, Okumura et al. 2017) | Yes | No | Unclear | Yes | Yes | Yes | 4/5 | High |
| Yilin Li (Li, Zhang et al. 2016) | Yes | Yes | Yes | Yes | Yes | Yes | 6/6 | Low |
| Katarina Kolostova (Kolostova, Matkowski et al. 2016) | Yes | Unclear | Unclear | Yes | Yes | No | 3/4 | UN |
| Yilin Li (Li, Gong et al. 2016) | Yes | Yes | Yes | Yes | Yes | No | 5/6 | High |
| H. Okabe (Okabe, Tsunoda et al. 2015) | Yes | Yes | Yes | Yes | Yes | Yes | 6/6 | Low |
| Su Jin Lee (Lee, Lee et al. 2015) | Yes | Yes | No | No | Yes | Yes | 4/6 | High |
| Kosei Toyoshima (Toyoshima, Hayashi et al. 2015) | Yes | Unclear | Unclear | No | Yes | No | 2/4 | High |
| Study | **Q1** | **Q2** | **Q3** | **Q4** | **Q5** | **Q6** | **Quality Score** | **Risk of bias?** |
| Yilin Li (Li, Zhang et al. 2014) | Yes | Unclear | No | Unclear | No | Unclear | 1/3 | High |
| Man Li  (Li, Zhang et al. 2014) | Yes | Yes | No | Yes | Unclear | No | 3/5 | High |
| Satoshi Matsusaka (Matsusaka, Chìn et al. 2010) | Yes | Yes | Yes | Yes | Yes | No | 5/6 | High |
| Baoguang Hu (Hu, Tian et al. 2020) | Yes | Yes | Yes | Yes | Yes | Yes | 6/6 | Low |
| Ilja Kubisch (Kubisch, de Albuquerque et al. 2015) | Yes | Yes | Yes | Yes | Yes | Yes | 6/6 | Low |
| Yoshikazu Uenosono (Uenosono, Arigami et al. 2013) | Yes | Yes | Yes | Yes | Yes | Yes | 6/6 | Low |
| Hiroaki Ito  (Ito, Inoue et al. 2012) | No | Yes | Yes | Yes | No | No | 3/6 | High |
| Yongpin Liu (Liu, Ling et al. 2017) | Yes | Yes | Yes | Yes | Yes | Yes | 6/6 | Low |
| Joon Hyung Jhi  (Jhi, Kim et al. 2021) | Yes | Yes | Yes | Yes | Yes | Yes | 6/6 | Low |
| Zhenlong Ye (Ye, Ding et al. 2019) | Yes | Yes | Yes | Yes | Yes | No | 5/6 | High |
| Qiyue Zhang (Zhang, Shan et al. 2018) | Yes | Yes | Yes | Yes | Yes | Yes | 6/6 | Low |
| Daniel Brungs (Brungs, Lynch et al. 2018) | Yes | No | No | No | Yes | No | 2/6 | High |
| L. Zheng (Zheng, Zou et al. 2017) | Yes | No | No | Yes | Yes | No | 3/6 | High |
| Hwa Mi Kang (Kang, Kim et al. 2017) | Yes | Yes | No | Yes | No | No | 3/6 | High |
| Study | **Q1** | **Q2** | **Q3** | **Q4** | **Q5** | **Q6** | **Quality Score** | **Risk of bias?** |
| Hiroaki Ito  (Ito, Sato et al. 2016) | Yes | Yes | Yes | Yes | Yes | No | 5/6 | High |
| Dandan Yuan (Yuan, Chen et al. 2015) | Yes | No | No | Unclear | Unclear | Unclear | 1/6 | High |
| M Iwatsuki (Iwatsuki, Toyoshima et al. 2013) | Yes | No | Yes | Yes | No | No | 3/6 | High |
| Pengjie Yu  (Yu, Zhu et al. 2022) | Yes | Yes | Yes | Yes | Yes | Yes | 6/6 | Low |

Table S1. Continue

Q1. Were adequate eligibility Criteria developed and applied?

Q2. Was the measurement of both exposure and outcome adequate?

O3. Was confounding adequately controlled for?

Q4. Was the follow-up complete and adequate in duration?

Q5. Are reports of the study free of suggestion of selective outcome reporting?

Q6. Was the study free of other problems that put it at a high risk of bias?
