# Supplementary material for: Investigating the role of circulating tumor cells in gastric cancer: a comprehensive systematic review and meta-analysis
Source: Clin Exp Med. 2024 Mar 30;24(1):59. doi: 10.1007/s10238-024-01310-6 (PMC10981629; doi:10.1007/s10238-024-01310-6)
Supplement: Supplementary file 5 — Supplementary file5 (DOCX 51 KB) [file 10238_2024_1310_MOESM5_ESM.docx]

Supplementary Table 2. Overall estimates of meta-analysis on the risk differences (RD) of the presence of CTCs in sampling time

| Outcomes | subgroups | N | RD (95% CI) | P value | I^2^ (%) | P heterogeneity | P heterogeneity between subgroups | Reference |
| --- | --- | --- | --- | --- | --- | --- | --- | --- |
| Before and after treatment | | 13 | -0.17 (-0.28, -0.06) | 0.002 | 89.0 | <0.001 | - | (39, 41, 42, 46, 47, 53, 54, 61, 62, 71, 75, 79, 81) |
| Marker | Epithelial | 10 | -0.12 (-0.25, 0.00) | 0.050 | 89.3 | <0.001 | 0.079 | (39, 41, 53, 54, 61, 62)  (71, 75, 79, 81) |
|  | Mesenchymal | 3 | -0.35 (-0.57, -0.13) | 0.002 | 82.8 | 0.003 |  | (42, 46, 47) |
| Detection | Cell search | 6 | -0.19 (-0.28, -0.10) | <0.001 | 66.7 | 0.010 | 0.888 | (41, 54, 61, 71, 79, 81) |
|  | Other | 7 | -0.17 (-0.37, 0.03) | 0.090 | 92.9 | <0.001 |  | (39, 42, 46, 47, 53, 62)  (75) |
| Treatment type | Surgery | 4 | -0.18 (-0.40, 0.03) | 0.098 | 92.1 | <0.001 | 0.928 | (39, 46, 54, 79) |
|  | Chemotherapy | 9 | -0.17 (-0.31, -0.03) | 0.016 | 88.7 | <0.001 |  | (41, 42, 47, 53, 61, 62, 71, 75, 81) |
| Presence of distance metastasis | Yes | 10 | -0.21 (-0.34, -0.07) | 0.002 | 90.1 | <0.001 | 0.139 | (41, 42, 46, 47, 53, 61, 71, 75, 79, 81) |
|  | No | 3 | -0.06 (-0.20, 0.08) | 0.409 | 69.6 | 0.037 |  | (39, 54, 62) |
| Presence of lymph node metastasis | Yes | 6 | -0.17 (-0.39, 0.05) | 0.134 | 93.6 | <0.001 | 0.959 | (41, 46, 53, 75, 79, 81) |
|  | No | 7 | -0.18 (-0.29, -0.06) | 0.002 | 80.3 | <0.001 |  | (39, 42, 47, 54, 61, 62, 71) |
| Risk of bias | Low | 9 | -0.17 (-0.33, -0.02) | 0.031 | 92.6 | <0.001 | 0.940 | (39, 42, 46, 47, 54, 61, 62, 75, 79) |
|  | High | 4 | -0.18 (-0.28, -0.06) | <0.001 | 0.0 | 0.738 |  | (41, 53, 71, 81) |
