# Supplementary material for: Investigating the role of circulating tumor cells in gastric cancer: a comprehensive systematic review and meta-analysis
Source: Clin Exp Med. 2024 Mar 30;24(1):59. doi: 10.1007/s10238-024-01310-6 (PMC10981629; doi:10.1007/s10238-024-01310-6)
Supplement: Supplementary file 6 — Supplementary file6 (DOCX 15 KB) [file 10238_2024_1310_MOESM6_ESM.docx]

Supplementary Table 3. Meta-regression for the effect of baseline characteristics

| Variable | Coefficient | SE | P-value |
| --- | --- | --- | --- |
| HR of OS | | | |
| Age | -0.04 | 0.02 | 0.064 |
| Male to female ratio | -0.01 | 0.08 | 0.895 |
| Follow-up duration | -0.003 | 0.005 | 0.451 |
| Distance metastasis | -0.22 | 0.28 | 0.441 |
| Lymph node metastasis | -0.003 | 0.21 | 0.988 |
| HR of PFS |  |  |  |
| Age | -0.03 | 0.04 | 0.381 |
| Male to female ratio | -0.05 | 0.14 | 0.709 |
| Follow-up duration | 0.002 | 0.02 | 0.944 |
| Distance metastasis | 0.02 | 0.48 | 0.970 |
| Lymph node metastasis | -0.13 | 0.37 | 0.738 |
| RD of before-after treatment |  |  |  |
| Age | -0.01 | 0.01 | 0.400 |
| Male to female ratio | -0.01 | 0.06 | 0.811 |
| Follow-up duration | -0.003 | 0.006 | 0.596 |
| Distance metastasis | -0.14 | 0.13 | 0.306 |
| Lymph node metastasis | 0.01 | 0.12 | 0.916 |
| RD of early vs advanced stages |  |  |  |
| Age | 0.01 | 0.01 | 0.211 |
| Male to female ratio | -0.12 | 0.07 | 0.117 |
| Distance metastasis | -0.18 | 0.15 | 0.224 |
| Lymph node metastasis | 0.02 | 0.14 | 0.885 |
| RD of intestinal vs diffuse stages |  |  |  |
| Age | 0.01 | 0.02 | 0.594 |
| Male to female ratio | 0.05 | 0.08 | 0.571 |
| Distance metastasis | 0.04 | 0.24 | 0.876 |
| Lymph node metastasis | 0.01 | 0.21 | 0.942 |
| RD of intestinal vs mixed stages |  |  |  |
| Age | 0.02 | 0.02 | 0.467 |
| Male to female ratio | 0.01 | 0.11 | 0.957 |
| Distance metastasis | 0.09 | 0.29 | 0.758 |
| Lymph node metastasis | 0.002 | 0.38 | 0.996 |
| RD of diffuse vs mixed stages |  |  |  |
| Age | -0.001 | 0.01 | 0.852 |
| Male to female ratio | -0.05 | 0.07 | 0.500 |
| Distance metastasis | 0.02 | 0.21 | 0.919 |
| Lymph node metastasis | 0.007 | 0.29 | 0.982 |
